# Tinea nigra: A series of three cases observed in Botucatu, São Paulo

**DOI:** 10.1590/0037-8682-0563-2020

**Published:** 2021-03-08

**Authors:** Flávia de Oliveira Valentim, Priscila Neri Lacerda, Gabriela Roncada Haddad

**Affiliations:** 1 Universidade Estadual Paulista, Faculdade de Medicina, Programa de Residência Médica em Dermatologia, Departamento de Infectologia, Dermatologia, Diagnóstico por Imagem e Radioterapia, Botucatu, SP, Brasil.; 2 Universidade Estadual Paulista, Faculdade de Medicina, Departamento de Infectologia, Dermatologia, Diagnóstico por Imagem e Radioterapia, Botucatu, SP, Brasil.

**FIGURE 1:**
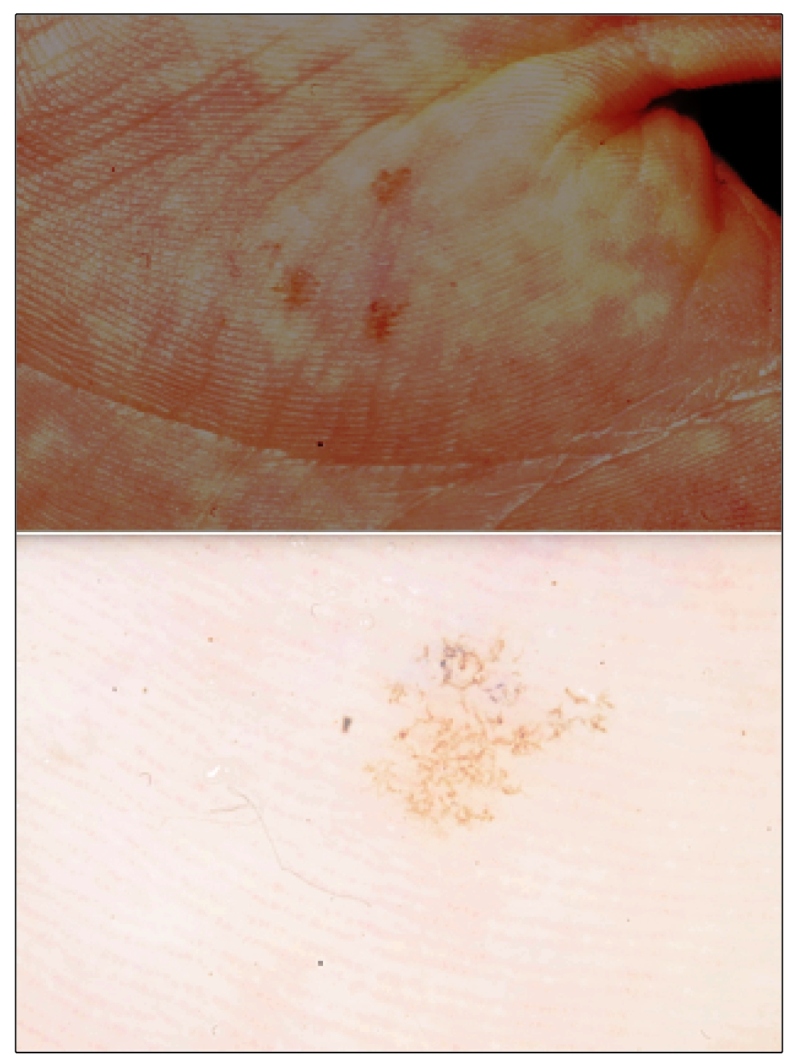
(A) Three hyperchromic macules on the thenar eminence of the left hand. (B) Dermoscopy showing thin, superficial, pigmented spikes, forming a reticular pattern.

**FIGURE 2:**
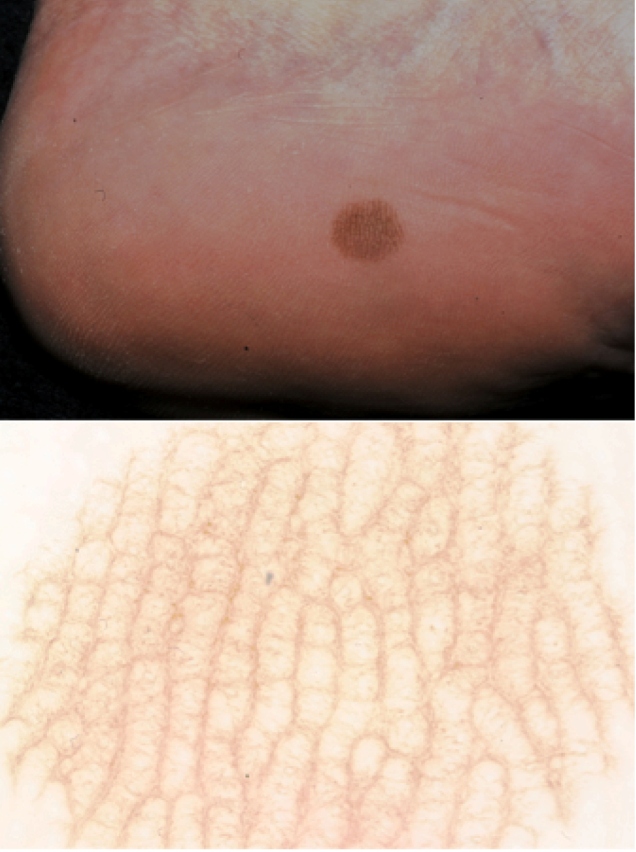
(A) Well-defined hyperchromic macula on the medial side of the left calcaneus (B) Dermoscopy showing thin, superficial, pigmented spicules, forming a pattern that did not follow the natural lines of the plantar surface.

**FIGURE 3:**
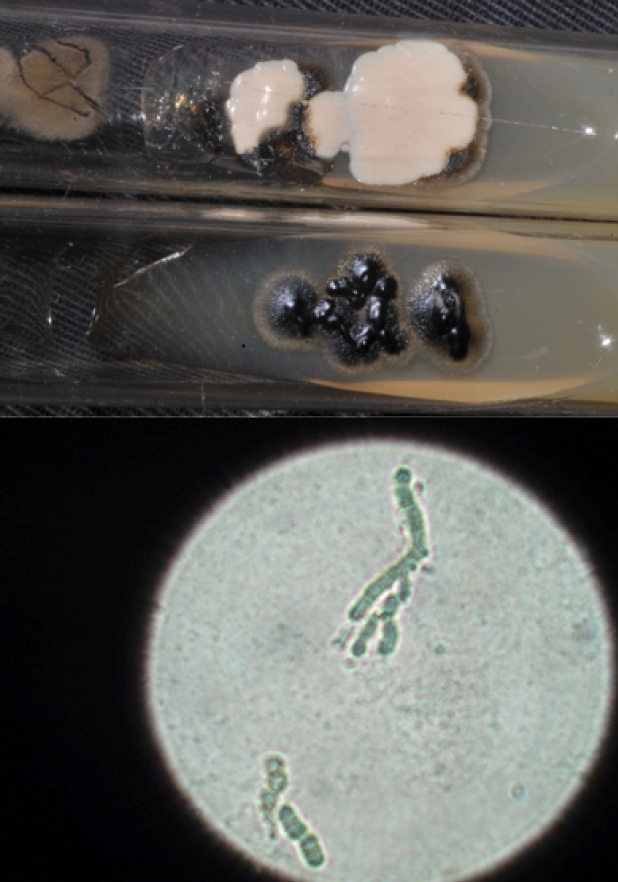
(A) Culture showing on the surface a membranous wrinkled colony, greyish-white coloring, and the reverse a black pigmented colony. (B) Direct mycological examination showing short, dark, septate, tortuous hyphae.

Tinea nigra is a rare, chronic, asymptomatic, and superficial keratophytosis caused by the dematiaceous fungus *Hortae werneckii*, which occurs mainly in tropical and subtropical regions^1^. We report images from a series of three cases observed in the city of Botucatu between 2011 and 2019 owing to the importance of this condition in the differential diagnosis of melanoma. The disease occurs more frequently in children and preferably affects the stratum corneum of palms and plants^1^. The clinical manifestation involves a brown-to-black macule with well-defined edges and precise limits, asymptomatic status, and no inflammation (Figure 1)^1^. There are defining characteristics in clinical analysis such as the presence of streaks; brownish or light brown or olive green superficial pigmentation; and regular distribution, concentrating it on both ridges and palmoplantar furrows (Figure 2)^2^. Dermoscopy shows blackish pigmentation, with a non-melanocytic, homogeneous, reticulate pattern^2^. The diagnosis is made by direct mycological examination with potassium hydroxide, microculture, and culture^3^. On direct mycological examination, short, dark (dematiaceous fungi), septate, tortuous hyphae were observed (Figure 3)^3^. Microculture showed brownish septate hyphae and brownish bicellular conidia^3^. Culture revealed a greyish-white, wrinkled, membranous surface colony, and the reverse presented with black pigmentation (Figure 3)^3^. It is important to recognize this disease early for proper treatment and to distinguish it from other melanocytic lesions with worse prognoses, such as melanoma.
